# The miR-1260b–NFAT5 axis regulates inflammasome priming and M1 macrophage polarization in periodontitis

**DOI:** 10.3389/fimmu.2026.1813346

**Published:** 2026-08-04

**Authors:** Chikako Hayashi, Takao Fukuda, Miyu Shida, Meng Xiao, Ziyu Wang, Naoaki Ryo, Masaaki Toyoda, Kentaro Kawakami, Takanori Shinjo, Tsukasa Aoki, Jinfeng Li, Mwannnes Ahmad, Takaharu Taketomi, Takeshi Uchiumi, Terukazu Sanui, Fusanori Nishimura

**Affiliations:** 1 Department of Periodontology, Division of Oral Rehabilitation, Faculty of Dental Science, Kyushu University, Fukuoka, Japan; 2 Dental and Oral Medical Center, Kurume University School of Medicine, Fukuoka, Japan; 3 Department of Clinical Chemistry and Laboratory Medicine, Graduate School of Medical Sciences, Kyushu University, Fukuoka, Japan

**Keywords:** inflammasome, macrophage, miR-1260b, NFAT5, periodontitis

## Abstract

**Introduction:**

Macrophage-driven inflammatory programs and NOD-like receptor family pyrin domain-containing 3 (NLRP3) inflammasome plays a critical role for periodontal tissue destruction; however, the transcriptional regulators that mediate these responses remain unclear. This study therefore examined the role of the microRNA-1260b (miR-1260b)–nuclear factor of activated T-cells 5 (NFAT5) axis in inflammatory macrophage responses relevant to periodontal disease.

**Methods:**

NFAT5 was identified as a candidate miR-1260b target using integrated bioinformatic screening and investigated through gain- and loss-of-function approaches in RAW264.7 macrophages. Inflammasome-related experiments were performed in J774A.1 macrophages using an LPS/ATP two-signal stimulation protocol. Further analyses included a ligature-induced mouse experimental periodontitis model with local NFAT5 small interfering RNA delivery, macrophage polarization assays in murine bone marrow-derived macrophages stimulated with lipopolysaccharide (LPS) and interferon-gamma (IFN-γ), and validation experiments in human peripheral blood mononuclear cell (PBMC)–derived macrophages.

**Results:**

Transfection with miR-1260b reduced NFAT5 expression and attenuated LPS-induced nuclear accumulation in macrophages. *In vivo* analyses showed increased NFAT5 expression in inflamed gingival tissues, whereas local NFAT5 knockdown was associated with reduced NLRP3 and interleukin-1 beta (IL-1β) expression. In J774A.1 macrophages, NFAT5 silencing under LPS/ATP stimulation reduced Nlrp3 and Il1b mRNA expression, together with lower NLRP3 and pro-IL-1β protein levels and reduced IL-1β levels in culture supernatants. Additional *in vitro* experiments demonstrated reduced M1-associated inflammatory gene expression following NFAT5 silencing. Flow cytometric analysis further showed a reduced proportion of CD86^+^ macrophage subsets after NFAT5 knockdown, accompanied by decreased pro-inflammatory cytokine production in both murine and human macrophage systems.

**Discussion:**

These findings identify NFAT5 as a key regulator of inflammasome priming that contributes to inflammatory macrophage polarization in periodontal inflammation and suggest the miR-1260b–NFAT5 axis as a potential target for host-modulatory approaches.

## Graphical Abstract

**Figure:**
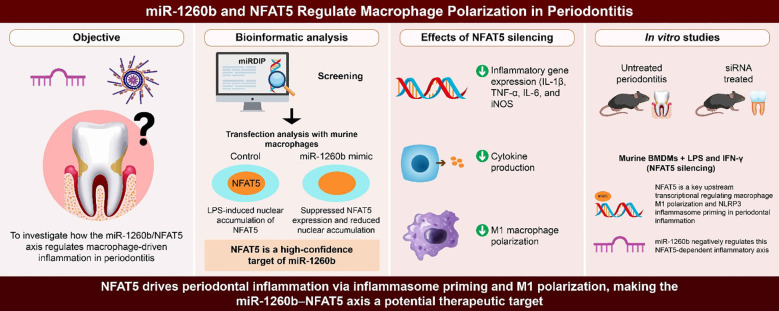

## 1 Introduction

Periodontitis is characterized by alveolar bone loss resulting from a chronic, dysregulated host inflammatory response to dysbiotic bacterial biofilms (1). NOD-like receptor family pyrin domain- containing 3 (NLRP3) inflammasomes serve as pivotal mediators in the pathogenesis of periodontitis (2). Inflammasomes are multiprotein complexes present in immune cells that detect microbial and danger signals, leading to the activation of caspase-1 and the maturation of pro-inflammatory cytokines, particularly interleukin (IL)-1β and IL-18 (3). Canonical activation of the NLRP3 inflammasome requires two sequential signals. The first signal (priming), typically provided by LPS, induces transcription of NLRP3 and pro-IL-1β, whereas the second signal, such as extracellular ATP, triggers inflammasome assembly and downstream inflammatory responses. The canonical LPS/ATP two-signal model is a standard approach for investigating NLRP3 inflammasome regulation (3–5). These cytokines subsequently mediate inflammation and bone resorption in periodontal tissues. NLRP3-deficient mice consistently exhibited impaired alveolar bone loss in a mouse experimental periodontitis model (6). Furthermore, increased NLRP3 inflammasome activity was strongly correlated with disease severity and tissue destruction in periodontitis (7).

Among immune and resident cells, macrophages are not only the primary source of IL-1β production, but also regulate the onset and resolution of inflammation in periodontal disease (8). Macrophages are broadly classified into two phenotypes: pro-inflammatory M1 (classically activated) and tissue remodeling M2 (alternatively activated) cells (9, 10). M1 macrophages are characterized by high expression of inflammatory markers, such as IL-1β, tumor necrosis factor alpha (TNF-α), inducible nitric oxide synthase (iNOS), and IL-6, as well as robust NLRP3 inflammasome activity that triggers acute inflammation. In contrast, M2 macrophages promote tissue repair and inflammation resolution by expression of IL-10, transforming growth factor-beta (TGF-β), and arginase-1 (11). Therefore, the modulation of the M1/M2 macrophage balance has emerged as a promising therapeutic approach for periodontitis.

Recent studies have demonstrated the potential use of extracellular vesicles (EVs) derived from mesenchymal stem cells (MSCs) for cell-free immunotherapy (12). MSC-EVs exert immunomodulatory effects on both adaptive (e.g., regulatory T cell induction) and innate immunity, particularly through macrophage polarization in inflammatory diseases (13). Specifically, emerging evidence suggests that optimization of microRNA (miRNA) content in MSC-EVs through preprocessing strategies holds considerable promise for significantly enhancing the therapeutic efficacy of MSC-EVs in specific diseases (14).

Nakao et al. (15) demonstrated that EVs derived from gingival tissue-derived MSCs (GMSCs) pre-treated with TNF-α enhanced macrophage polarization toward an anti-inflammatory (M2) phenotype, suggesting that this negative feedback loop could serve as a therapeutic strategy. In a mouse experimental periodontitis model, these EVs were shown to significantly promote wound healing and inhibit periodontal bone loss compared with control EVs (15). Hayashi et al. (16) further reported that miR-1260b, a TNF-α–inducible miRNA, regulates osteoclastogenesis. However, the direct association between miR-1260b and macrophage phenotype regulation, as well as the underlying molecular mechanisms, has not yet been fully elucidated.

Nuclear factor of activated T-cells 5 (NFAT5), also known as tonicity-responsive enhancer-binding protein (TonEBP), is a unique transcription factor initially identified for its role in the osmotic stress response (17). Recent studies have indicated that NFAT5 is involved in immune regulation beyond osmotic stress, particularly in macrophage activation and inflammasome signaling (18, 19), and associated with NLRP3 inflammasome–related pathways (20). Periodontal tissues often exhibit hyperosmotic conditions during inflammation (21), and NFAT5 has been identified as an autoantigen associated with periodontal diseases (22). Furthermore, recent studies have indicated a potential NFAT5–NLRP3 regulatory axis in non-periodontal settings across multiple cell types, including endothelial cells (20) and microglia (23); however, its role in macrophage-mediated periodontal inflammation remains unclear. Given that macrophages exhibit high NLRP3 expression among hematopoietic cells and that NLRP3 critically enhances the M1 phenotype (24), elucidating the NFAT5–NLRP3 connection in macrophages may provide novel therapeutic insights. This study investigated whether NFAT5 functions as a mediator involved in miR-1260b–dependent regulation of macrophage NLRP3/IL-1β responses.

## 2 Materials and methods

### 2.1 Bioinformatic analysis

Target genes of miR-1260b were predicted using the mirDIP database (https://ophid.utoronto.ca/mirDIP/). Prediction settings were defined as an Integrated Score > 0.25, with the score class restricted to the top 5% confidence tier to identify high-confidence targets relevant to inflammatory signaling.

### 2.2 Experimental animals and ethics statement

Male C57BL/6NCrSlc mice (8 weeks old) were purchased from Japan SLC, Inc. (Hamamatsu, Japan) and maintained under specific pathogen-free conditions. All animal procedures were approved by the Animal Care and Use Committee of Kyushu University (Protocol Number: A25-085-0) and performed in accordance with the ARRIVE guidelines. Commercially available de-identified human CD14^+^ monocytes were obtained from Lonza Group Ltd. (Basel, Switzerland) and differentiated into macrophages *in vitro*. The authors did not collect samples directly from human participants and had no access to identifiable donor information. Therefore, institutional review board approval and informed consent were not required for this study.

### 2.3 Preparation of NFAT5 siRNA–polyethylenimine nanoparticles

Linear polyethylenimine nanoparticles optimized for *in vivo* delivery (*in vivo*-jetPEI; Polyplus-Transfection SA, Illkirch-Graffenstaden, France) were used to deliver siRNAs. Stealth RNAi duplexes targeting murine NFAT5 were pooled (MSS225642, MSS225643, MSS285158; Invitrogen Life Technologies/Thermo Fisher Scientific, Carlsbad, CA, USA). A Stealth RNAi Negative Control Duplex (Medium GC; Invitrogen) was used as control. Each siRNA was dissolved in sterile 5% (w/v) glucose to a concentration of 20 μM. The siRNA and PEI were mixed and incubated for 15 min at room temperature to form complexes. For each injection, 10 μL containing 50 pmol of siRNA was prepared.

### 2.4 Ligature-induced mouse experimental periodontitis model and local siRNA administration

Experimental periodontitis was induced in mice, as previously described (25). Each experimental group consisted of five mice (n = 5 per group). A 6–0 silk ligature (Akiyama Medical MFC Co., Tokyo, Japan) was placed around the right maxillary second molar on day 0 and maintained for 7 days. Mice were randomly assigned to siCtrl or siNFAT5 groups. Immediately after ligature placement (day 0), siRNA–PEI-NPs were injected into the palatal gingiva adjacent to the ligated molar using a 33-gauge Hamilton microsyringe (Hamilton Company, Reno, NV, USA). Additional injections were administered on day 3. Sample size (n = 5 per group) was determined based on prior experience with this model and commonly used group sizes in comparable studies, as well as ethical considerations to minimize animal use.

### 2.5 Histology and immunofluorescence staining

Maxillae were fixed in 4% (w/v) paraformaldehyde for 24 h, demineralized in an EDTA-based solution (Osteosoft; Merck KGaA, Darmstadt, Germany) for 72 h, and embedded in paraffin. Sections were deparaffinized, rehydrated, and subjected to antigen retrieval. Non-specific binding was blocked using G-Block (Genostaff, Tokyo, Japan) for tissue sections or Blocking One (Nacalai Tesque, Kyoto, Japan) for cultured cells. NFAT5 was detected using anti-NFAT5 rabbit polyclonal antibody (1:100; ab3446; Abcam, Cambridge, UK) followed by Goat Anti-Rabbit Recombinant Secondary Antibody, CoraLite Plus 488 (Proteintech, Tokyo, Japan). Nuclei were counterstained with SlowFade Diamond Antifade Mountant with DAPI (Thermo Fisher Scientific; S36964). Images were acquired using a confocal laser-scanning microscope (Nikon AX; Nikon, Tokyo, Japan). Fluorescence intensity was quantified using ImageJ (NIH, Bethesda, MD, USA). Confocal imaging conditions were based on previously described procedures (26). The fluorescence intensity of NFAT5 in individual cells was quantified using ImageJ software (National Institutes of Health, Bethesda, MD, USA). Regions of interest (ROIs) corresponding to nuclei and cytoplasm were manually defined, background fluorescence was subtracted, and the mean fluorescence intensity was calculated for each ROI.

### 2.6 Cell culture and reagents

RAW264.7 (ATCC TIB-71; Manassas, VA, USA) and J774A.1 (JCRB9108; JCRB Cell Bank, Osaka, Japan) murine macrophages were cultured in high-glucose DMEM (4.5 g/L) supplemented with 2 mM L-glutamine, 1 mM sodium pyruvate (FUJIFILM Wako, Osaka, Japan), 10% (v/v) heat-inactivated FBS, and 0.1% (v/v) penicillin-streptomycin. For bone marrow-derived macrophages (BMDMs), bone marrow cells were collected from the femurs and tibias of C57BL/6 mice, subjected to red blood cell lysis, and cultured in α-MEM (FUJIFILM Wako) containing 10% heat-inactivated FBS, 1% penicillin-streptomycin, and 25 ng/mL recombinant mouse macrophage colony-stimulating factor (M-CSF; BioLegend, San Diego, CA, USA) for 7 days, with medium replacement on day 3. Human CD14+ monocytes (Lonza Group Ltd., Basel, Switzerland) were obtained commercially as de-identified primary human cells and subsequently differentiated into macrophages in RPMI-1640 (Nacalai Tesque) supplemented with 10% heat-inactivated FBS, 2 mM L-glutamine, 1% sodium pyruvate, 1% non-essential amino acids, 1% penicillin-streptomycin, and 25 ng/mL recombinant human M-CSF for 6–7 days. Because these commercially obtained human cells were de-identified and were not collected directly from human participants by the authors, institutional review board (IRB) approval and informed consent were not required for this study. Reagents used for stimulation included recombinant mouse interferon gamma (IFN-γ) (BioLegend), Lipopolysaccharide (LPS) from Escherichia coli O55:B5 (Sigma-Aldrich, St. Louis, MO, USA) and ATP (Sigma-Aldrich).

### 2.7 *In vitro* transfection and stimulation conditions

For functional studies, cells were transfected using HiPerFect Transfection Reagent (Qiagen, Hilden, Germany) according to the manufacturer’s protocol. For miRNA overexpression, 20 pmol miRCURY LNA miR-1260b mimic (Qiagen) or negative control cel-miR-39-3p mimic (Qiagen) was used. For NFAT5 knockdown, Stealth RNAi duplexes targeting human NFAT5 (HSS116542; Invitrogen) or pooled murine NFAT5 siRNAs (MSS225642, MSS225643, MSS285158; Invitrogen) were used with Stealth RNAi Negative Control Duplex (Medium GC; Invitrogen). For the inflammasome experiments, J774A.1 cells were seeded in 6-well plates and transfected with 25 pmol siRNA or miRNA mimic per well using 6 μL HiPerFect. After 6 h, the medium was replaced, and cells were cultured in DMEM supplemented with 10% FBS and penicillin-streptomycin. Stimulation was started 24 h after the initial transfection. For qRT-PCR, J774A.1 cells were stimulated with LPS (100 ng/mL) for 3 h followed by ATP (5 mM) for 45 min. For western blotting and ELISA, J774A.1 cells were stimulated with LPS (100 ng/mL) for 6 h followed by ATP (5 mM) for 45 min. J774A.1 macrophages were stimulated using a canonical LPS/ATP two-signal protocol for NLRP3 inflammasome activation. Inflammatory/M1 stimulation conditions were standardized across *in vitro* systems as follows: RAW264.7 macrophages were stimulated with E. coli LPS (O55:B5) at 10 ng/mL plus IFN-γ at 25 ng/mL for 24 h. For M1 polarization, BMDMs and human macrophages were stimulated with E. coli LPS (O55:B5) at 10 ng/mL plus IFN-γ at 25 ng/mL for 24 h, as widely used in inflammatory macrophage activation studies (27, 28).

### 2.8 Quantitative real-time PCR

Total RNA was extracted using ISOGEN II (Nippon Gene, Tokyo, Japan). First-strand cDNA was synthesized using PrimeScript RT Master Mix (Takara Bio, Otsu, Japan). qRT-PCR was performed using KAPA SYBR FAST qPCR Kit (Master Mix, 2× Universal; Roche Diagnostics, Basel, Switzerland) on a LightCycler 96 system (Roche). Cycling conditions were 95 °C for 20 s, followed by 40 cycles of 95 °C for 10 s, 60 °C for 20 s, and 72 °C for 1 s. miRNA quantification was performed as previously described (29). Primer sequences are listed in **Supplementary Table 1**.

### 2.9 Western blot analysis

Cultured cells were washed with PBS and lysed in Passive Lysis 5× Buffer (Promega, Madison, WI, USA). Nuclear and cytoplasmic protein fractions were isolated using the LysoPure Nuclear and Cytoplasmic Extractor Kit (FUJIFILM Wako). Proteins were separated by SDS-PAGE and transferred to PVDF membranes according to standard procedures, as previously described (30). Membranes were probed with anti-NFAT5 (1:1000; ab3446; Abcam), anti-IL-1β (3A6) mouse monoclonal antibody (1:1000; 12242; Cell Signaling Technology) for detection of the pro-IL-1β band, anti-NLRP3 (1:1000; clone D4D8T; 15101; Cell Signaling Technology), anti-Lamin B1 (1:1000; 1298-1-AP; Proteintech), and anti-β-actin (1:1000; clone 13E5; Cell Signaling Technology). HRP-conjugated anti-rabbit IgG secondary antibody (1:2000; Cell Signaling Technology) was used for detection. Immunoreactive bands were visualized using an Amersham ImageQuant 800 imaging system (Cytiva, Tokyo, Japan) and analyzed using ImageJ software (National Institute of Health, Bethesda, MA, USA). In J774A.1 cells, NFAT5 was analyzed in the nuclear fraction, whereas NLRP3 and pro-IL-1β were analyzed in whole-cell lysates. Lamin B1 and β-actin were used as the respective loading controls.

### 2.10 Enzyme-linked immunosorbent assay

IL-1β concentrations in culture supernatants were quantified using the Quantikine Mouse IL-1β/IL-1F2 immunoassay kit (R&D Systems, Minneapolis, MN, USA; MLB00C) according to the manufacturer’s instructions. All assays were performed in triplicate for each experimental condition.

### 2.11 Flow cytometric analysis

Flow cytometry was performed using a BD FACSLyric cytometer (Becton Dickinson, Franklin Lakes, NJ, USA). Human macrophages were blocked with Human TruStain FcX (BioLegend) and stained with PE-anti-human CD86 and APC-anti-human CD206 (BioLegend). Murine BMDMs were blocked with TruStain FcX anti-mouse CD16/32 (BioLegend) and stained with PE/Cy7-anti-mouse F4/80, PE-anti-mouse CD86, and APC-anti-mouse CD206 (all BioLegend). Corresponding isotype controls (BioLegend) were used for gating.

### 2.12 Statistical analysis

Data are presented as mean ± SD. Statistical analyses were performed using GraphPad Prism version 10 (GraphPad Software, La Jolla, CA, USA). Data distribution was assessed using the Shapiro–Wilk test. Parametric tests were applied to normally distributed data, whereas non-parametric tests (Mann–Whitney U test for two-group comparisons or Kruskal–Wallis test followed by Dunn’s multiple comparison test for multiple-group comparisons, as appropriate) were used when normality assumptions were not met. A P-value < 0.05 was considered statistically significant.

### 2.13 Data availability and ethics statement

The raw data supporting the conclusions of this article will be made available by the authors upon reasonable request. All animal experiments were approved by the Animal Care and Use Committee of Kyushu University (protocol number: A25-085-0). For human samples, commercially obtained de-identified cryopreserved cells were used in accordance with the supplier’s ethical framework.

## 3 Results

### 3.1 miR-1260b suppresses NFAT5 expression and nuclear localization in LPS-stimulated macrophages

To identify candidate miR-1260b targets relevant to inflammatory macrophage programming, an integrated target prediction approach using the mirDIP database was performed and prioritized genes with plausible role in inflammatory signaling (**Table 1**). NFAT5 was identified as a high-confidence candidate (integrated score = 0.2557; “High” category, top 1/3) and was selected for downstream validation based on its predicted involvement in inflammatory stress signaling, macrophage polarization, and NLRP3/IL-1β–associated responses; other predicted targets included ATF6β, Wnt5a, TLR4, RANKL, and NFATc1.

**Table 1:** Canidate target genes of miR-1260b identified by mirDIP analysis.

Target gene	Gene name	Biological function	NCBI gene ID	Integrated score	Confidence rank
ATF6B	Activating transcription factor 6 beta	Transcription factor involved in the unfolded protein response during ER stress	1388	0.6303	Very high; Top 1%
WNT5A	Wnt family member 5A	Non-canonical Wnt signaling	7474	0.2991	High; Top 5%
NFAT5	Nuclear factor of activated T-cells 5	Osmotic stress response and immune regulation	10725	0.2557	High; Top 1/3
RANKL (TNFSF11)	Tumor necrosis factor superfamily member 11 (TNFSF11)	Essential for osteoclast formation	8600	0.1877	Medium
TLR4	Toll-like receptor 4	LPS sensor and pro-inflammatory cytokine induction	7099	0.1204	Medium
NFATC1	Nuclear factor of activated T-cells, cytoplasmic 1	Master regulator of osteoclast differentiation	4772	0.1019	Medium

UPR, unfolded protein response; LPS, lipopolysaccharide; RANKL, receptor activator of nuclear factor-κB ligand.

Candidate target genes of miR-1260b were identified by mirDIP analysis and prioritized based on integrated prediction scores and biological relevance to inflammatory signaling, immune regulation, and osteoclast-related pathways.

**Figure 1:**
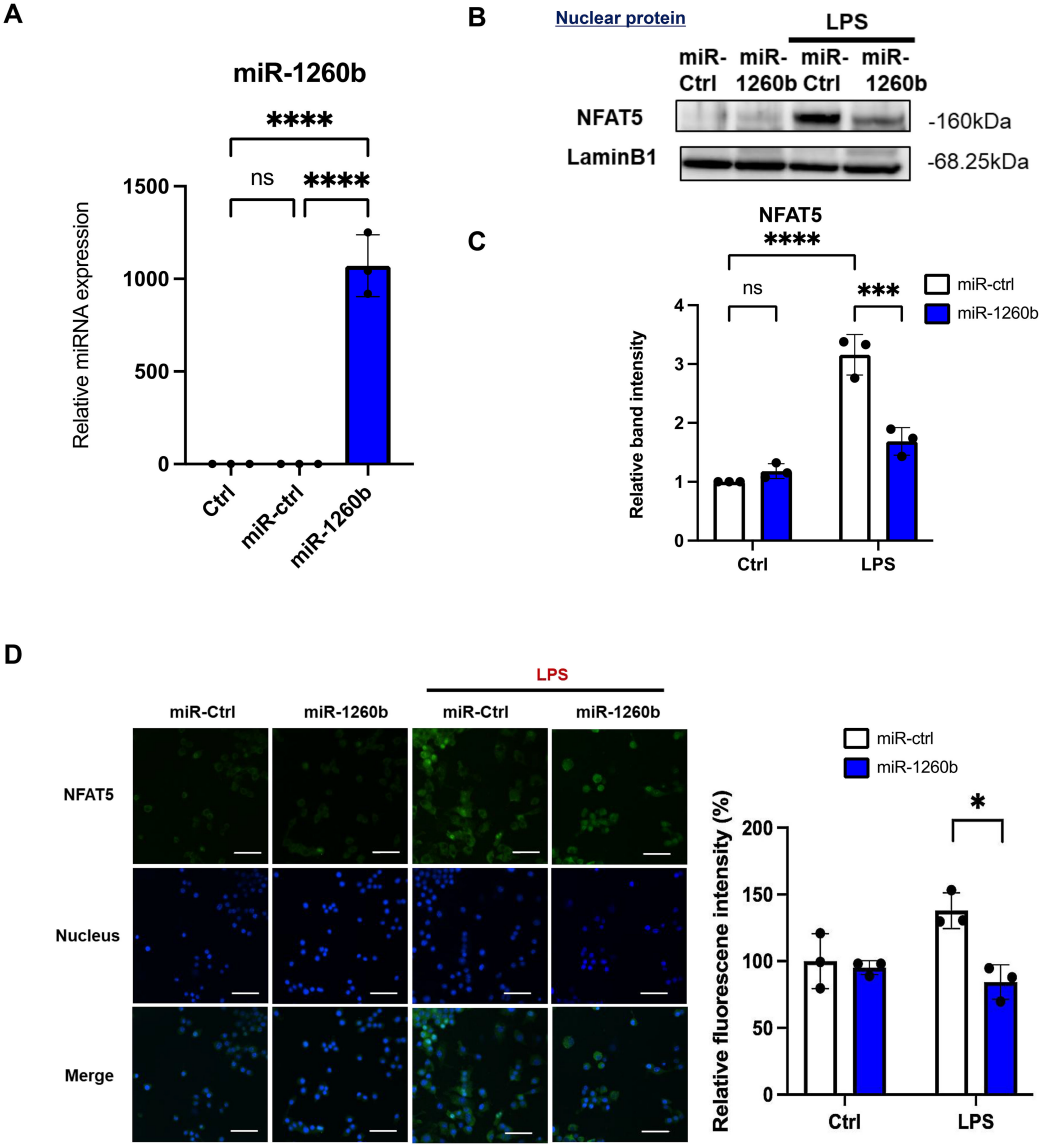
Experimental validation of miR-1260b-mediated NFAT5 regulation in macrophages. **(A)** The transfection efficiency of miR-1260b in RAW264.7 macrophages was confirmed by qRT-PCR. n=3. **(B)** Western blot of nuclear NFAT5 protein in RAW264.7 cells transfected with miR-Ctrl or miR-1260b mimic, followed by LPS stimulation (100 ng/mL, 24 h). Lamin B1 was used as the control for nuclear protein. **(C)** Densitometric quantification of nuclear NFAT5. n=3. **(D)** Confocal immunofluorescence images showing NFAT5 (green) and nuclei (DAPI, blue). Intensity of NFAT5 fluorescence was measured in each group. n=3 Scale bar = 20 μm. *p < 0.05, ***p < 0.001, ****p < 0.0001. Statistical analyses were performed using parametric or non-parametric tests, as appropriate, based on data distribution, followed where applicable by Tukey’s or Dunn’s multiple comparison test.

To validate the functional regulatory relationship between miR-1260b and NFAT5 under inflammatory conditions, RAW264.7 murine macrophages were transfected with miR-1260b mimic followed by LPS stimulation. Western blot analysis of nuclear protein fractions showed that LPS stimulation increased nuclear NFAT5 protein levels in miR-Ctrl-transfected cells, whereas transfection with the miR-1260b mimic reduced nuclear NFAT5 accumulation (**Figures 1B, C**). Immunofluorescence microscopy further provided spatial resolution of these effects, revealing that while LPS stimulation led to pronounced nuclear localization of NFAT5 in control cells, miR-1260b mimic-transfected cells exhibited significantly diminished nuclear NFAT5 signals (**Figure 1D**). These results suggest that miR-1260b negatively regulates NFAT5 expression in macrophages.

### 3.2 NFAT5 is upregulated in a mouse experimental periodontitis model and promotes NLRP3/IL-1β expression in gingival tissues

**Figure 2:**
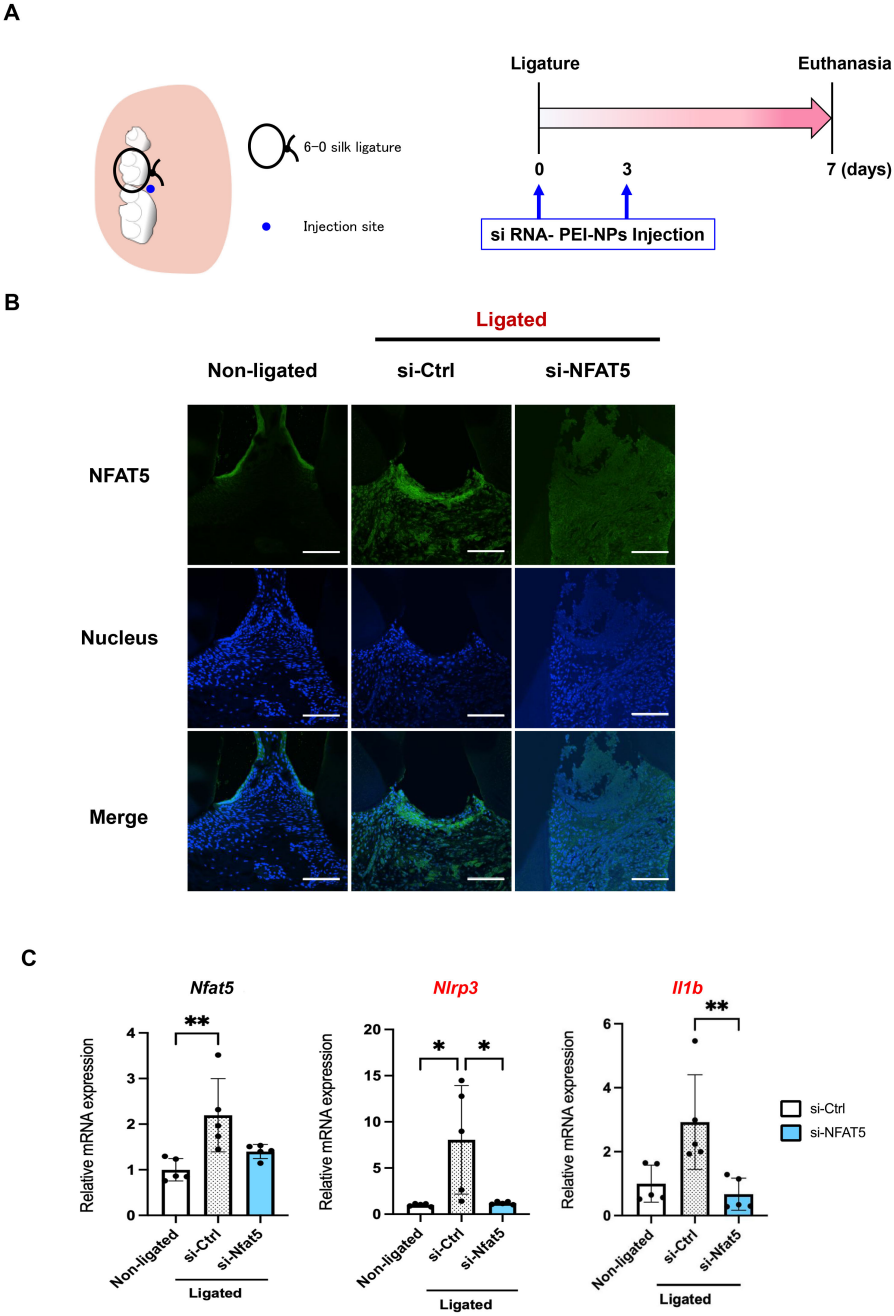
NFAT5 upregulation in a ligature-induced mouse experimental periodontitis model and effects of siRNA-mediated knockdown on inflammatory gene expression. **(A)** Experimental design for ligature-induced mouse experimental periodontitis model and siRNA-PEI-NP administration. A 6–0 silk ligatures were placed on day 0, siRNA injections on days 0 and 3, and tissue harvest on day 7. **(B)** Immunofluorescence staining of NFAT5 (green) in periodontal tissues. Nuclei stained with DAPI (blue). Scale bar = 50 μm. **(C)** qRT-PCR analysis of *Nfat5*, *Nlrp3*, and *Il1b* mRNA in mice gingival tissues. n=5 per group. *p < 0.05, **p < 0.01. Statistical analyses were performed using parametric or non-parametric tests, as appropriate, based on data distribution, followed where applicable by Tukey’s or Dunn’s multiple comparison test.

To investigate the pathophysiological involvement of NFAT5 in periodontal disease, a ligature-induced mouse experimental periodontitis model was used (**Figure 2A**). NFAT5 expression was higher in gingival tissues from ligated sites than in those from non-ligated controls, as demonstrated using immunofluorescence staining (**Figure 2B**). Local administration of NFAT5 siRNA (si-NFAT5) effectively reduced NFAT5 signals in periodontal tissues relative to the control siRNA.

Given the emerging evidence implicating NFAT5 with NLRP3 inflammasome activation in various inflammatory contexts, whether NFAT5 knockdown influenced inflammasome-related gene expression was examined in periodontal tissues. qRT-PCR analysis revealed that si-NFAT5 significantly decreased the mRNA levels of *Nlrp3* (the inflammasome sensor protein) and *Il1b* (the key inflammasome-dependent cytokine) in ligated gingival tissues compared with control siRNA treatment (**Figure 2C**). These results indicate that NFAT5 is induced during periodontal inflammation and contributes to the transcriptional upregulation of NLRP3 and IL-1β *in vivo*.

### 3.3 NFAT5 knockdown attenuates priming-associated inflammasome responses under LPS/ATP stimulation in J774A.1 macrophages

**Figure 3:**
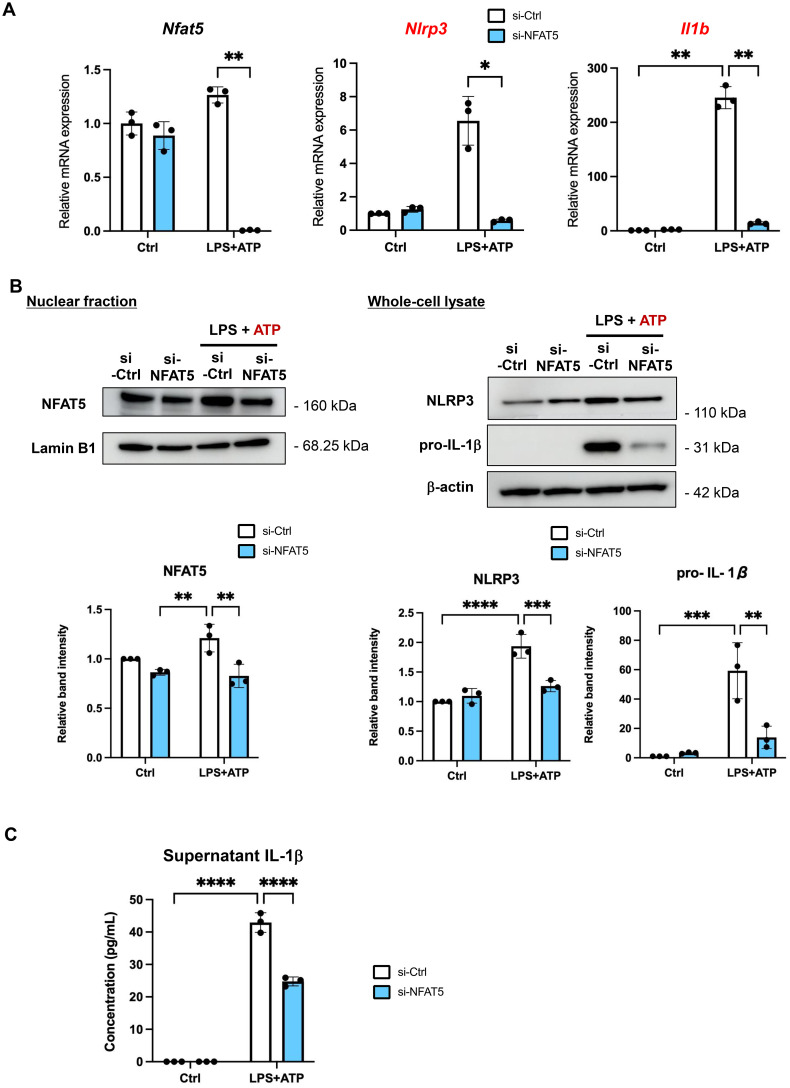
NFAT5 silencing attenuates priming-associated inflammasome responses under LPS/ATP stimulation in J774A.1 macrophages. **(A)** qRT-PCR analysis of Nfat5, Nlrp3, and Il1b expression in J774A.1 macrophages transfected with si-Ctrl or si-NFAT5 under control or LPS/ATP-stimulated conditions. For qRT-PCR, cells were stimulated with LPS (100 ng/mL) for 3 h followed by ATP (5 mM) for 45 min. **(B)** Representative western blot analysis of NFAT5 in the nuclear fraction and of NLRP3 and pro-IL-1β in whole-cell lysates from J774A.1 macrophages transfected with si-Ctrl or si-NFAT5 under control or LPS/ATP-stimulated conditions. For western blotting, cells were stimulated with LPS (100 ng/mL) for 6 h followed by ATP (5 mM) for 45 min. Lamin B1 and β-actin were used as loading controls for the nuclear fraction and whole-cell lysates, respectively. Densitometric quantification is shown below. **(C)** ELISA measurement of IL-1β levels in cell culture supernatants from J774A.1 macrophages transfected with si-Ctrl or si-NFAT5 under control or LPS/ATP-stimulated conditions. n=3. **p < 0.01, ***p < 0.001, ****p < 0.0001. Statistical analyses were performed using parametric or non-parametric tests, as appropriate, based on data distribution, followed where applicable by Tukey’s or Dunn’s multiple comparison test.

To further examine the involvement of NFAT5 under a two-signal stimulation protocol, loss-of-function studies were performed in J774A.1 macrophages. After transfection with si-Ctrl or si-NFAT5, cells were stimulated with LPS followed by ATP. qRT-PCR analysis showed that LPS/ATP stimulation increased Nfat5, Nlrp3, and Il1b expression in si-Ctrl cells, whereas si-NFAT5 attenuated the induction of Nlrp3 and Il1b (**Figure 3A**). Consistent with this, western blot analysis showed reduced nuclear NFAT5 levels and lower NLRP3 and pro-IL-1β protein levels in si-NFAT5-transfected cells under LPS/ATP stimulation (**Figure 3B**). ELISA analysis showed that IL-1β levels in culture supernatants were lower in si-NFAT5-transfected cells than in si-Ctrl-transfected cells (**Figure 3C**). These findings support a role for NFAT5 primarily in inflammasome priming under LPS/ATP stimulation. Similar tendencies were also observed in J774A.1 macrophages transfected with miR-1260b (**Supplementary Figure 1**), and supportive data from the previous RAW264.7 LPS-based system are presented in **Supplementary Figure 2**.

### 3.4 NFAT5 silencing inhibits M1 macrophage polarization in bone marrow-derived macrophages

**Figure 4:**
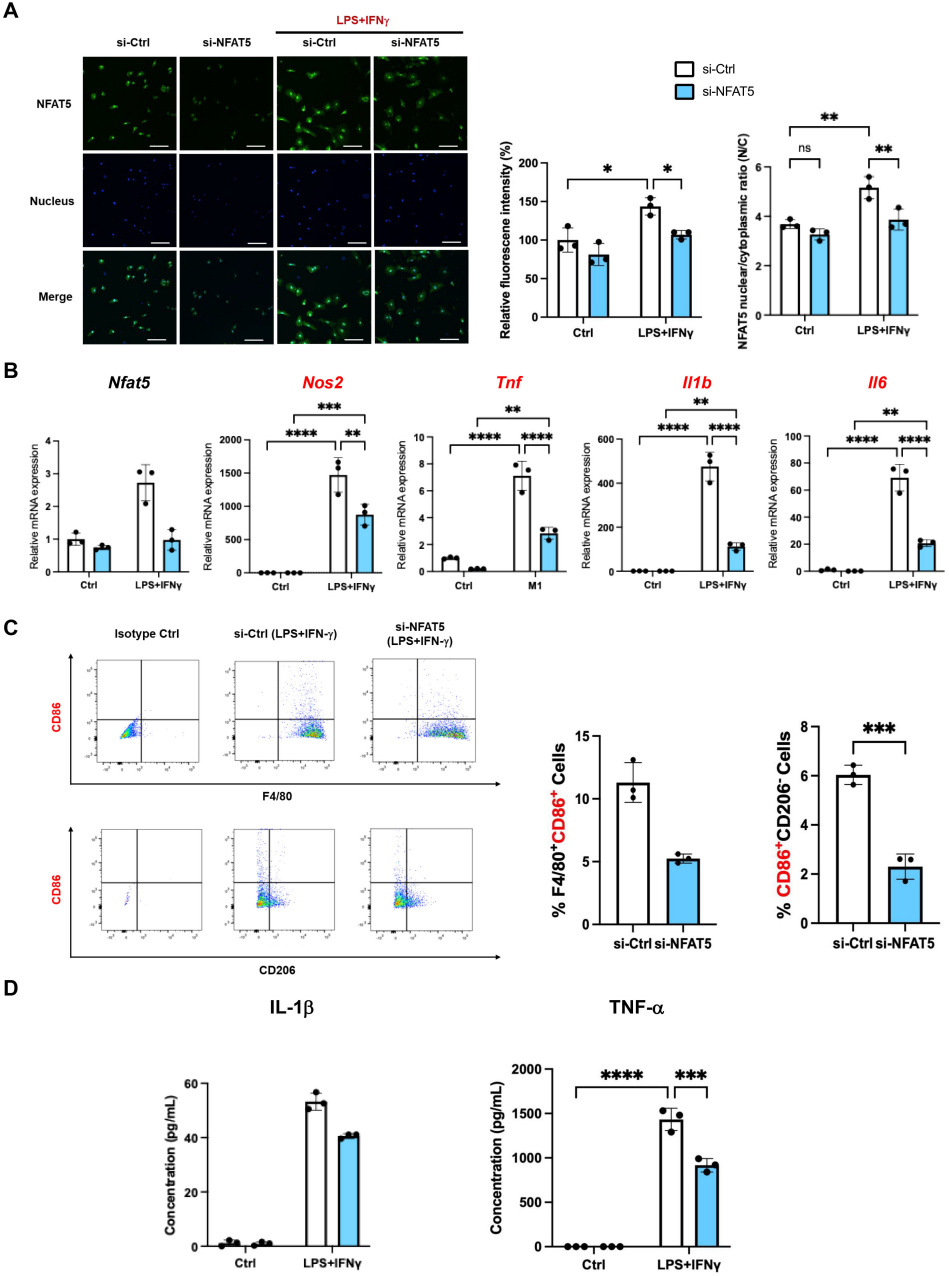
NFAT5 knockdown attenuates M1 macrophage polarization in primary bone marrow-derived macrophages. **(A)** Confocal microscopy of NFAT5 in BMDMs transfected with si-Ctrl or si-NFAT5, followed by LPS+IFN-γ stimulation (left). Scale bar = 20 μm. Expression and the cellular localization of NFAT5 in each sample were measured in each group (right). **(B)** qRT-PCR analysis of *Nfat5* and M1 markers (*Nos2, Tnf*, *Il1b*, and *Il6*). n=3. **(C)** Flow cytometry gating strategy for F4/80^+^ macrophages showing CD86 (M1) and CD206 (M2) expression. Representative dot plots for isotype control, si-Ctrl, and si-NFAT5 transfected cells under M1 (LPS+IFN)-inducing conditions (left). Quantification of F4/80^+^CD86^+^ and CD86^+^CD206^-^ populations (right). n=3. **(D)** ELISA measurement of IL-1β and TNF-α levels in culture supernatants. *p < 0.05, **p < 0.01, ***p < 0.001, ****p < 0.0001. Statistical analyses were performed using parametric or non-parametric tests, as appropriate, based on data distribution, followed where applicable by Tukey’s or Dunn’s multiple comparison test.

Given the pathogenic contribution of M1 macrophages in periodontal tissue destruction, the regulation of inflammatory polarization by NFAT5 was examined in primary murine bone marrow–derived macrophages (BMDMs). BMDMs were transfected with control siRNA (si-Ctrl) or si-NFAT5 and subjected to classical M1 polarization induced by combined stimulation with LPS and IFN-γ. Immunofluorescence imaging confirmed efficient NFAT5 silencing, showing significantly attenuated nuclear NFAT5 translocation in si-NFAT5-transfected cells compared with si-Ctrl (**Figure 4A**). qRT-PCR analysis demonstrated that LPS+IFN-γ stimulation robustly induced the expression of *NFAT5* and prototypical M1-associated genes, including *Il1b, Tnfa, Il6*, and *Nos2* (iNOS), in si-Ctrl-transfected BMDMs, whereas these transcriptional responses were significantly attenuated in si-NFAT5–transfected cells (**Figure 4B**).

Flow cytometric analysis within the F4/80^+^ macrophage population further revealed that NFAT5 silencing decreased the proportion of F4/80^+^ CD86^+^ cells (a canonical M1 fraction) and reduced the frequency of the CD86^+^ CD206^−^ subset compared with si-Ctrl (**Figure 4C**). Consistent with these immunophenotypic changes, NFAT5 knockdown also reduced the production of M1-type inflammatory cytokines (IL-1β and TNF-α) compared with si-Ctrl (**Figure 4D**). Collectively, these findings suggest that NFAT5 promotes M1 polarization and inflammatory cytokine production during LPS/IFN-γ–driven activation.

### 3.5 NFAT5-dependent regulation of macrophage polarization is conserved in human monocyte-derived macrophages

**Figure 5:**
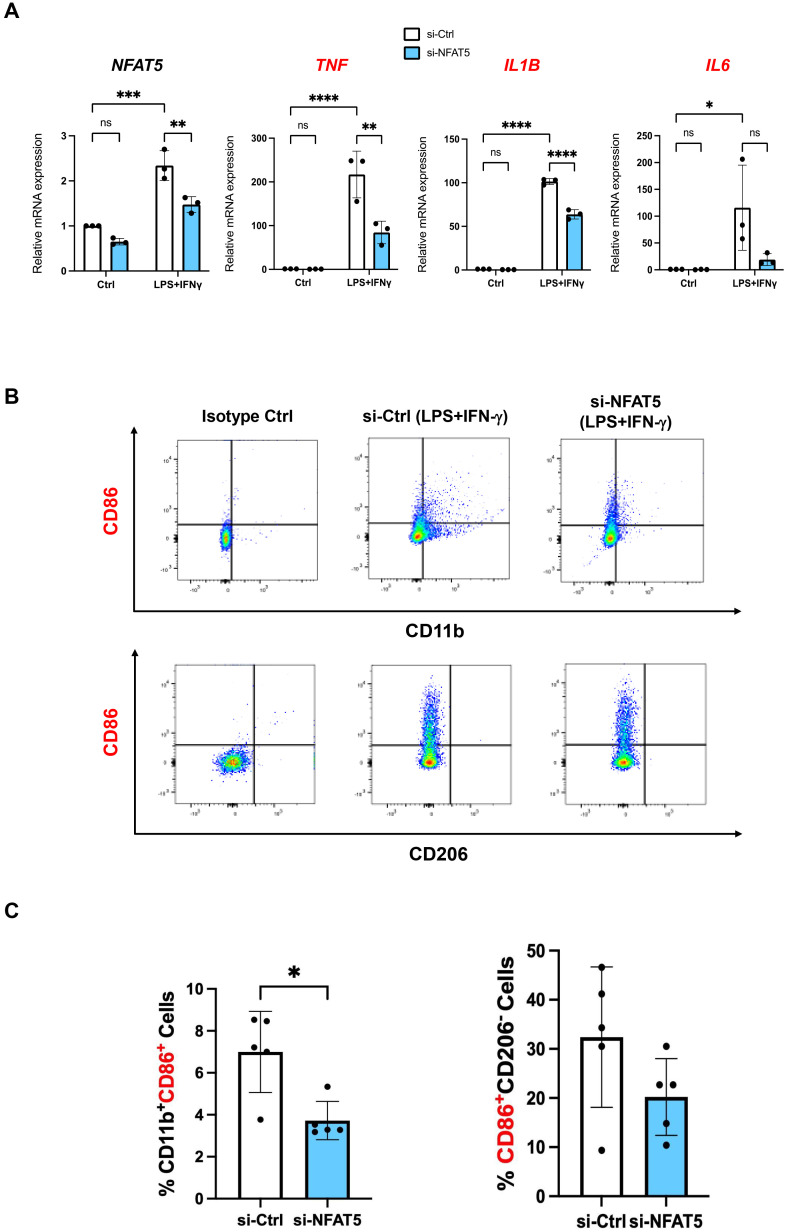
Cross-species validation of NFAT5-dependent macrophage polarization regulation in human PBMC-derived macrophages. **(A)** qRT-PCR analysis of NFAT5 and M1-associated cytokine expressions (TNF-α, IL-1β, IL-6) in human macrophages transfected with si-Ctrl or si-NFAT5, followed by LPS+IFN-γ stimulation. n=3. ***p < 0.001, ****p < 0.0001. **(B)** Flow cytometry gating strategy for CD11b^+^ macrophages showing CD86 (M1) and CD206 (M2) expression. Representative dot plots for isotype control, si-Ctrl, and si-NFAT5 transfected cells under M1 (LPS+IFN)-inducing conditions. **(C)** Quantification of CD11b^+^CD86^+^ and CD86^+^CD206^-^ populations (right). n=5. *p < 0.05, **p < 0.01, ***p < 0.001, ****p < 0.0001. Statistical analyses were performed using parametric or non-parametric tests, as appropriate, based on data distribution, followed where applicable by Tukey’s or Dunn’s multiple comparison test.

To establish translational relevance, investigations were extended to primary human macrophages derived from PBMCs. PBMC-derived macrophages were transfected with si-Ctrl or si-NFAT5 and subjected to M1-inducing stimulation. As observed in murine macrophages, M1 induction upregulated NFAT5 expression, whereas NFAT5 knockdown significantly suppressed the expression of M1-associated cytokines (*TNF*, *IL1B*, and *IL6*) (**Figure 5A**). Flow cytometric analysis further showed that NFAT5 knockdown significantly reduced the proportion of CD11b^+^CD86^+^ cells, while the frequency of CD86^+^CD206^−^ cells showed a decreasing tendency compared with the si-Ctrl condition (**Figures 5B, C**). These results indicate that NFAT5 supports the maintenance of an M1-skewed phenotype in human macrophages under inflammatory stimulation.

## 4 Discussion

Macrophage-driven inflammatory responses are central to periodontal pathogenesis, and the present study identifies NFAT5 as a central transcription factor associated with macrophage polarization and inflammasome priming during periodontal inflammation. Periodontitis is a chronic inflammatory disease in which persistent activation of innate immune responses critically contributes to connective tissue destruction and alveolar bone loss (1, 2, 7). Among the innate immune cells, macrophages play a central role in periodontal inflammation by integrating microbial stimuli and tissue-derived stress signals into coordinated inflammatory programs, including NLRP3 inflammasome activation and polarization toward pro-inflammatory phenotypes (8) (31).

The results of this study suggest that NFAT5 emerges as a key transcription factor associated with macrophage M1 polarization and NLRP3 inflammasome priming in periodontal inflammation, while miR-1260b negatively regulates this NFAT5-dependent inflammatory axis. Among the predicted miR-1260b target genes (**Table 1**), NFAT5 was selected for downstream validation because it is associated with inflammatory stress signaling, macrophage polarization, and NLRP3/IL-1β–related responses—key processes in periodontal immunopathology. The J774A.1 experiments further supported regulation at the level of inflammasome priming, consistent with NFAT5-dependent changes in Nlrp3 and Il1b expression, NLRP3 and pro-IL-1β protein levels, and IL-1β levels in culture supernatants under LPS/ATP stimulation (**Figure 3**).

Inflammasome activation in periodontal lesions is well recognized as a transcriptionally regulated process rather than solely a downstream inflammatory event (3, 5). The NLRP3 inflammasome promotes IL-1β–dependent inflammatory amplification and tissue destruction through tightly regulated priming and activation processes (6, 32, 33). In J774A.1 macrophages, NFAT5 knockdown under LPS/ATP stimulation reduced Nlrp3 and Il1b expression, lowered NLRP3 and pro-IL-1β protein levels, and decreased IL-1β levels in culture supernatants. Since activation-stage events such as caspase-1 cleavage or mature IL-1β processing were not directly examined, the present findings are best interpreted as reflecting regulation mainly at the inflammasome priming stage.

Although the present *in vitro* experiments were conducted under standard culture conditions without deliberate manipulation of osmolarity, previous studies have demonstrated that periodontal inflammation is associated with increased osmotic pressure in periodontal tissues (18). In addition, clinical studies have reported osmotic alterations in the gingival crevicular fluid of periodontitis patients, including elevated Na^+^ and K^+^ concentrations that correlate with clinical disease severity (34). Given that NFAT5 is a tonicity-responsive transcription factor, the periodontal microenvironment may provide both inflammatory and osmotic cues that modulate NFAT5-dependent signaling *in vivo*. However, because osmolarity was not monitored or experimentally controlled in the present study, it remains unclear whether the observed miR-1260b–NFAT5 regulatory axis is dependent on, or independent of, osmotic stress. Future studies using controlled hyperosmotic conditions will be required to clarify this issue.

These findings support a model in which miR-1260b suppresses NFAT5 expression and nuclear localization in macrophages. In this context, miR-1260b was found to reduce NFAT5 expression and nuclear accumulation, whereas NFAT5 knockdown under LPS/ATP stimulation decreased NLRP3 expression, Il1b transcription, pro-IL-1β protein levels, and IL-1β levels in culture supernatants. These observations indicate that the miR-1260b–NFAT5 axis modulates inflammasome priming through NFAT5-dependent regulation of inflammatory gene expression.

As shown in **Figure 3**, NFAT5 knockdown reduced Nlrp3 and Il1b induction, lowered NLRP3 and pro-IL-1β protein levels, and decreased IL-1β levels in culture supernatants under LPS/ATP stimulation. The use of the canonical LPS/ATP two-signal model in ASC-competent J774A.1 macrophages is consistent with the interpretation that NFAT5 primarily regulates the priming phase of the NLRP3 inflammasome (3–5). Consistent with this, NFAT5 knockdown was associated with reduced M1-associated inflammatory gene expression and a decreased proportion of CD86-positive inflammatory macrophages in primary BMDMs (**Figure 4**). **Supplementary Figure 1** showed similar changes in J774A.1 macrophages transfected with miR-1260b, and **Supplementary Figure 2** showed a similar overall tendency in the previous RAW264.7 LPS-based system. Because activation-stage events such as caspase-1 cleavage and mature IL-1β processing were not directly examined, the present findings are best interpreted as supporting a role for NFAT5 mainly in inflammasome priming.

Based on the previous studies implicating NFAT5 (TonEBP) in stress-responsive immune regulation (19, 35), this concept was extended to periodontal inflammation. The present data suggest that NFAT5 is involved in the regulation of NLRP3 and IL-1β expression, supporting a model in which NFAT5 appears to contribute to the inflammatory signaling that promotes macrophage activation during periodontal inflammation (20, 22, 23). Marked upregulation of NFAT5 expression in ligature-induced periodontal inflammation was observed, and local knockdown of NFAT5 significantly suppressed NLRP3 and IL-1β expression *in vivo* (**Figure 2C**). In the same ligature-induced mouse experimental periodontitis model, it was previously demonstrated that the local administration of a miR-1260b mimic significantly attenuates alveolar bone loss (16). Integrating previous phenotypic findings with the current supporting data suggests that miR-1260b functions as a regulatory factor that modulates inflammatory macrophage responses in periodontal disease.

Beyond inflammasome priming, NFAT5 may also influence macrophage polarization dynamics. In addition to its role in inflammasome priming, NFAT5 contributes to macrophage polarization. NFAT5 silencing attenuated M1-associated gene expression and reduced the proportion of CD86^+^ macrophages in both murine and human macrophages (**Figures 4**, **5**), suggesting that NFAT5 actively promotes a pro-inflammatory M1 phenotype. This is consistent with the established evidence on inflammatory macrophage polarization in periodontal disease (9, 10, 36). Given that M1 polarization and NLRP3 inflammasome activation mutually reinforce each other through feed-forward inflammatory loops (37), NFAT5 may contribute to the maintenance of a pro-inflammatory macrophage phenotype by influencing both inflammasome priming and polarization. Previous studies have primarily focused on individual inflammatory mediators. In contrast, our findings indicate regulation at the transcriptional level. Our results from this study support a model in which NFAT5 acts as a central regulator of multiple inflammatory pathways involved in macrophage activation. Consistent with this, NFAT5 appears to regulate both inflammasome priming and macrophage polarization, contributing to transcriptional regulation of inflammatory macrophage responses. Accordingly, targeting key transcriptional regulators such as NFAT5 may offer a strategy to simultaneously modulate multiple inflammatory pathways rather than focusing on single effector molecules such as individual cytokines.

Current host-modulatory strategies for periodontitis have largely focused on individual inflammatory mediators such as individual cytokines. Targeting key transcriptional regulators such as NFAT5 may allow simultaneous attenuation of multiple inflammatory responses, including cytokine production, inflammasome competence, and polarization-associated gene programs. Moreover, functional evidence was provided demonstrating that miR-1260b suppresses both NFAT5 expression and nuclear localization (**Figure 1**). This observation suggests that miR-1260b constrains NFAT5 transcriptional activity, consistent with established models of microRNA-mediated post-transcriptional regulation (38–40). These findings extend our previous observations on the therapeutic relevance of miR-1260b in a ligature-induced mouse experimental periodontitis model (16), in which local injection of miR-1260b mimic clearly suppressed alveolar bone resorption as compared with control miRNA-injected group. These data support the development of EV-based delivery of miR-1260b to target NFAT5-dependent inflammatory pathways, in line with international guidelines for EV research (41).

Although the present study does not determine whether NFAT5 directly binds regulatory elements of inflammasome-related genes in macrophages *in vivo*, previous studies suggest that NFAT5 can enhance inflammatory transcriptional programs through promoter engagement and cooperation with canonical inflammatory transcription factors such as NF-κB, supporting its role as a key regulator of inflammasome competence (20, 42). This study had several limitations. Although the data in this study implicated NFAT5 in inflammasome regulation and macrophage polarization, macrophage-specific genetic approaches are necessary to establish cell-intrinsic NFAT5 causality *in vivo*. Future studies are required to determine whether NFAT5 also modulates other inflammasome pathways or influences inflammasome-related inflammatory responses in non-macrophage periodontal cell populations. Collectively, our findings indicate that NFAT5 contributes to inflammatory macrophage responses through regulation of inflammasome priming and macrophage polarization. Further studies will be required to define the precise transcriptional mechanisms involved and to clarify whether NFAT5 directly regulates inflammasome-associated genes *in vivo*. If confirmed, targeting this pathway may provide a potential strategy for host-modulatory approaches in periodontal disease.

## 5 Conclusions

This study identifies NFAT5 as a key regulator of inflammasome priming and inflammatory macrophage polarization in periodontitis. miR-1260b suppressed NFAT5 expression and nuclear localization, whereas NFAT5 silencing reduced NLRP3 expression, Il1b transcription, pro-IL-1β protein levels, and IL-1β levels in culture supernatants. These findings suggest that the miR-1260b–NFAT5 axis contributes to the regulation of macrophage-driven inflammation and may represent a potential target for host-modulatory therapy in periodontitis. However, because activation-stage inflammasome events were not directly examined, further studies are needed to determine whether NFAT5 also contributes to the activation phase of the NLRP3 inflammasome.

## Data Availability

The original contributions presented in the study are included in the article/**Supplementary Material**. Further inquiries can be directed to the corresponding authors.
