# Supplementary material for: The miR-1260b–NFAT5 axis regulates inflammasome priming and M1 macrophage polarization in periodontitis
Source: Front Immunol. 2026 Aug 4;17:1813346. doi: 10.3389/fimmu.2026.1813346 (PMC13481538; doi:10.3389/fimmu.2026.1813346)
Supplement: Supplementary file 1 [file Table1.docx]

Supplementary Material

**Supplementary Table S1.**

Primer sequence used for quantitative RT-PCR

| Gene | Forward primer | Reverse primer |
| --- | --- | --- |
| *human TNF-α* | 5'-**CCCAGGGACCTCTCTCTAATCA**-3' | 5'-**GCTTGAGGGTTTGCTACAACATG**-3' |
| *human IL-1β* | 5'-**ACGATGCACCTGTACGATCA**-3' | 5'-**TCTTTCAACACGCAGGACAG**-3' |
| *human IL-6* | **5′-ATGAGGAGACTTGCCTGGTG-3′** | **5′-GGTCAGGGGTGGTTATTGCAT-3′** |
| *human GAPDH* | **5'-ATCAAGAAGGTGGTGAAGCAGG-3'** | **5'-GTCATACCAGGAAATGAGC-3'** |
| *mouse Nfat5* | **5'-GAGGGGTGTGGATTGGAATCT-3’** | **5’-CTGGTGCTCATGTTACTGAAGTT-3’** |
| *mouse Nlrp3* | **5’-ATTACCCGCCCGAGAAAGG-3’** | **5’-CATGAGTGTGGCTAGATCCAAG-3’** |
| *mouse Il1b* | **5’-GAAGAAGAGCCCATCCTCTG-3’** | **5’-TCATCTCGGAGCCTGTAGTG-3’** |
| *mouse Nos2* | **5’-GAGACAGGGAAGTCTGAAGCAC-3’** | **5’-CCAGCAGTAGTTGCTCCTCTTC-3’** |
| *mouse Tnf* | **5’-GACAGTGACCTGGACTGTGG-3’** | **5’-TGAGACAGAGGCAACCTGAC-3’** |
| *mouse Il6* | **5’-TACCACTTCACAAGTCGGAGGC-3’** | **5’-CTGCAAGTGCATCATCGTTGTTC-3’** |
| *mouse 18s rRNA* | **5'-GCTTAATTTGACTCAACACGGGA-3'** | **5'-AGCTATCAATCTGTCAATCCTGTC -3’** |

**Supplementary Figure S1. miR-1260b attenuates priming-associated inflammatory responses under LPS/ATP stimulation in J774A.1 macrophages.** (**A**) Representative western blot analysis of NLRP3 and pro-IL-1β in J774A.1 macrophages transfected with miR-Ctrl or miR-1260b under control or LPS/ATP-stimulated conditions. β-actin was used as a loading control. Densitometric quantification of NLRP3 and pro-IL-1β band intensities is shown on the right. Cells were stimulated with LPS (100 ng/mL) for 6 h followed by ATP (5 mM) for 45 min. (**B**) ELISA measurement of supernatant IL-1β levels in J774A.1 macrophages transfected with miR-Ctrl or miR-1260b under control or LPS/ATP-stimulated conditions. n=3. **p < 0.01, ***p < 0.001, ****p < 0.0001. Statistical analyses were performed using parametric or non-parametric tests, as appropriate, based on data distribution, followed where applicable by Tukey’s or Dunn’s multiple comparison test.

**Supplementary Figure S2. NFAT5 silencing reduces NLRP3 protein levels and IL-1β production in LPS-stimulated RAW264.7 macrophages.** **(A)** Confocal immunofluorescence showing NFAT5 knockdown efficiency in RAW264.7 cells transfected with si-Ctrl or si-NFAT5, followed by LPS stimulation. NFAT5 (green), nuclei (DAPI, blue). Scale bar = 20 μm. **(B)** Western blot analysis of nuclear NFAT5 and whole-cell NLRP3. Lamin B1 and β-actin were used as loading controls. **(C)** qRT-PCR analysis of *Il1b* mRNA expression (left) and ELISA measurement of secreted IL-1β levels in culture supernatants (right). n=3. *p < 0.05, **p < 0.01, ****p < 0.0001. Statistical analyses were performed using parametric or non-parametric tests, as appropriate, based on data distribution, followed where applicable by Tukey’s or Dunn’s multiple comparison test.
